# Investigations of Long-Acting Formulations in Children, Adolescents, and Pregnant Women: A Systematic Review

**DOI:** 10.3390/pharmaceutics17010113

**Published:** 2025-01-15

**Authors:** Lynn Bertagnolli, Zhengyi Deng, Melissa Davy-Rothwell, Elaine J. Abrams, Charles Flexner, Ethel D. Weld

**Affiliations:** 1 Division of Clinical Pharmacology, Department of Medicine, School of Medicine, The Johns Hopkins University, Baltimore, MD 21287, USA; 2 School of Medicine, Stanford University, Palo Alto, CA 94305, USA; zdeng18@stanford.edu; 3 Department of Health, Behavior, and Society, The Johns Hopkins Bloomberg School of Public Health, Baltimore, MD 21205, USA; mdavey1@jhu.edu; 4 ICAP, Mailman School of Public Health, Columbia University, New York, NY 10032, USA; eja1@cumc.columbia.edu; 5 Department of Pediatrics, Vagelos College of Physicians & Surgeons, Columbia University, New York, NY 10032, USA; 6 Division of Infectious Diseases, Department of Medicine, School of Medicine, The Johns Hopkins University, Baltimore, MD 21287, USA

**Keywords:** long-acting therapeutics, special populations, pediatrics, pregnancy

## Abstract

**Background/Objectives:** Long-acting and extended-release drug delivery strategies have greatly improved treatment for a variety of medical conditions. Special populations, specifically infants, children, young people, and pregnant and postpartum women, could greatly benefit from access to these strategies but are often excluded from clinical trials. We conducted a systematic review of all clinical studies involving the use of a long-acting intramuscular injection or implant in infants, children, young people, and pregnant and postpartum people. **Methods:** Pubmed, Embase, and Cochrane Library trials were searched. Studies published from 1980 through 2018 were included. After abstract review and duplication removal, full-text articles were obtained for further review, reviewed by two independent reviewers, and disagreements were resolved by a third reviewer. **Results:** a total of 101 studies of long-acting therapeutics were completed in these populations, and most (80%) of these had a sample size of <100 individuals. Therapeutics for only a small pool of indications were examined in these studies, with 72% of the studies investigating hormonal contraception or other types of hormonal treatments. Only 9.3% of the studies in children and 16.7% of the studies in pregnant people collected any pharmacokinetic (PK) data. **Conclusions:** Long-acting formulations may behave differently (both pharmacokinetically and pharmacodynamically) in childhood, adolescence, and pregnancy as compared to non-pregnant adulthood. Therefore, it is imperative to increase and improve upon the studies investigating long-acting formulations in order to close the knowledge gap and improve care and treatment in these special populations.

## 1. Introduction

Long-acting and extended-release drug delivery strategies have a strong track record for improving outcomes in the treatment and prevention of a variety of medical conditions. Some of the first long-acting innovations were long-acting reversible contraceptives (LARCs), such as implantable uterine devices (IUD) and contraceptive implants. Because these methods of contraception are “forgettable” to the user, i.e., independent of adherence, LARC methods have been shown to be highly successful as compared to daily oral contraceptive pills and are approximately 20 times as effective in preventing conception as strategies such as pills, patches, and rings [1]. Approaches to osteoporosis, chronic schizophrenia, and pain control have similarly been radically altered by the availability of such formulations [2,3,4,5,6].

Long-acting strategies with less than daily dosing frequency have improved adherence and clinical outcomes for nearly every clinical domain where they have been studied [7,8,9]. Despite the potential benefits of such formulations for vulnerable populations like children, young people, and pregnant and postpartum people, these groups are generally excluded from clinical drug trials, especially in the pre-approval phase, and we hypothesized that long-acting therapeutics would be no exception to this rule [10,11,12,13]. In addition, partially as a consequence of the perceived risk of teratogenicity of investigational drugs, there has been a long-standing trend toward exclusion of women of reproductive potential from the drug development process, or a requirement that they discontinue study participation entirely should they become pregnant [14,15]. There is often very little knowledge about the safety and pharmacokinetics of drugs in pregnancy at the time of drug approval; only 12 drugs have been endorsed by the Food and Drug Administration (FDA) for use in pregnancy at the time of approval. Most of those agents were for pregnancy- or delivery-specific indications [16,17]. The advent of long-acting drugs introduces both a conundrum and an opportunity, as it is often not possible to discontinue the long-acting drug at the time an individual becomes pregnant—it has already been administered and will still be present in their systemic circulation for months of pregnancy. This has laid a rational foundation for allowing participants who become pregnant while enrolled in studies of long-acting agents to continue both the drug and their participation in study.

Because of inadequate or non-existent safety and efficacy data, the majority of drugs prescribed to children are used off-label. In a study by Shah et al., it was found that in 78.7% of pediatric hospital discharges in a large inpatient resource utilization database, at least one drug was prescribed off-label [18]. This discrepancy is partially attributable to the lack of dispersible, pediatric-friendly oral formulations and low pharmaceutical company motivation to conduct trials in children. The development and manufacture of pediatric-specific formulations is a costly endeavor, so often is not pursued, particularly as long-term financial return is ultimately perceived by manufacturers to be limited. Another contributing factor is the worry (among parents, clinicians, regulators, sponsors, and even children themselves) that the risks of enrolling children and young people in clinical trials does not outweigh the benefits that the clinical trial can provide to an individual participant or to society. Any inherent risks are then shifted over into clinical practice, where drugs are used in special populations in whom there are no safety or efficacy data. The consequences of this reality remain largely undocumented and unheeded.

As one recent example, the approval of long-acting injectable cabotegravir and rilpivirine for HIV treatment, and LA-cabotegravir for HIV prevention [19,20], has led policymakers, healthcare providers, clinical investigators, patient advocacy groups, and community representatives to call for increased inclusion and access of pregnant people with HIV to novel long-acting technologies [17,21,22,23]. However, until recently, most phases of clinical trials for novel HIV therapeutics (including long-acting ones) have excluded pregnant—or potentially pregnant—people, partially due to the perception of enhanced risk of fetal harm from novel therapeutics.

Given the overall lack of inclusion of children and pregnant women in clinical research, we were interested in existing data regarding the safety and efficacy of long-acting therapeutics, for any indication, in children, young people, and pregnant and postpartum women. We undertook a systematic review of long-acting therapeutics that were specifically studied in infants, children, or pregnant/postpartum people, to better characterize the gaps specific to these populations. This effort may serve as a resource to clinicians and interested stakeholders and should also inform future research efforts in these key populations.

## 2. Methods

We analyzed clinical trials and post-hoc analyses of clinical trials completed after 1980 and prior to May 2018. Studies were included if they contained information on long-acting treatments in infants/children, defined as from birth to 12 years old, young people, defined as 10–24 years old, and pregnant people. For the purposes of this analysis, we focused on long-acting treatments that were dosed less frequently than every 2 weeks.

### 2.1. Data Sources and Search Strategy

Pubmed, Embase, and Cochrane Library trials were searched to identify articles meeting the search criteria. The full list of search terms used for each database can be found in the Supplementary Materials.

### 2.2. Study Selection

Five investigators independently reviewed study abstracts against inclusion and exclusion criteria. Studies were included if they were prospective clinical trials in any phase and/or post-hoc analyses of one trial. Studies investigating long-acting treatments, specifically intramuscular injections and implants, were included if they researched the therapy in children, young people, or pregnant and postpartum women. Exclusion criteria included studies of vaccines, studies of limited-duration anesthesia/analgesia, studies of treatment for postpartum hemorrhage, studies examining pain/infection related to surgery and labor, insulin studies, studies of abortion and ectopic pregnancy, studies of in vitro fertilization, studies involving animals, studies that exclusively had an adult sample (18+ years) without a focus on adolescents and youth, articles without an abstract, and articles not written in English.

### 2.3. Data Extraction and Quality Assessment

Discrepancies or disagreements about the inclusion of studies based on their abstract were resolved by a third investigator. After abstract review and duplication removal, full-text articles were obtained for further review. Full-text articles were reviewed by two independent reviewers and disagreements were resolved by a third reviewer.

### 2.4. Data Synthesis and Analysis

Detailed information on the demographic characteristics of the study population (race/ethnicity, age, sex), treatment (drug name, dosage, frequency, duration, and method of delivery, toxicity, side effects), main results, and conclusion was then extracted from each of the remainingstudies. A qualitative synthesis of this extracted information was then performed.

## 3. Results

**Figure 1:**
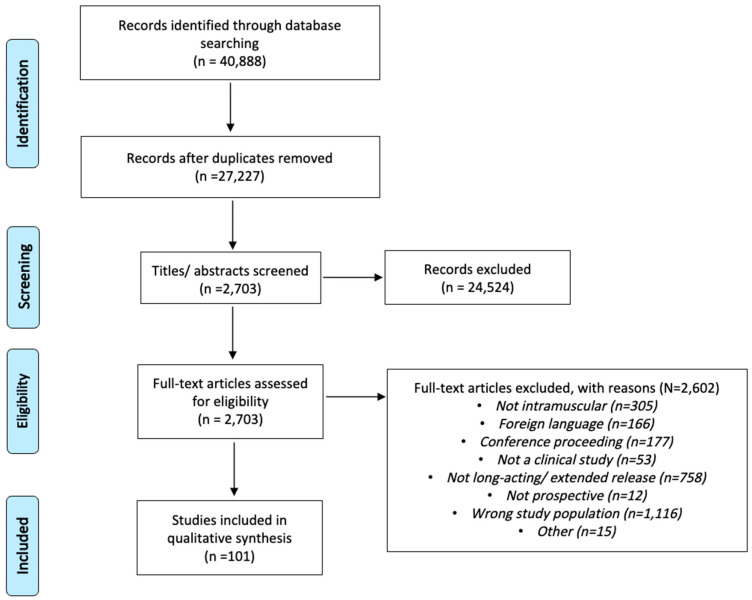
Preferred reporting items for systematic reviews and meta-analyses (PRISMA) diagram.

Using database searches, a total of 40,888 records were found using specified search terms (Supplementary Tables S2–S4). Of these, 13,661 records were excluded due to duplication, leaving 27,227 unique studies that were reviewed against inclusion and exclusion criteria. Subsequently, 2703 studies were selected and obtained from databases for full-text review. Of the studies that underwent full-text review, a total of 101 studies were found to meet inclusion and exclusion criteria. The PRISMA diagram (Figure 1) depicts the study process and flow. (Per PRISMA 2020 statement: an updated guideline for reporting systematic reviews, BMJ 2021; 372: n71 [24]).

Of the 101 studies identified after full-text review, 95 involved long-acting treatments in infants, children, and/or young people, three studies involved long-acting treatments in pregnant women, and three studies involved long-acting treatments in postpartum women. These studies are summarized in Table 1.

### 3.1. Distribution of Sample Sizes

**Figure 2:**
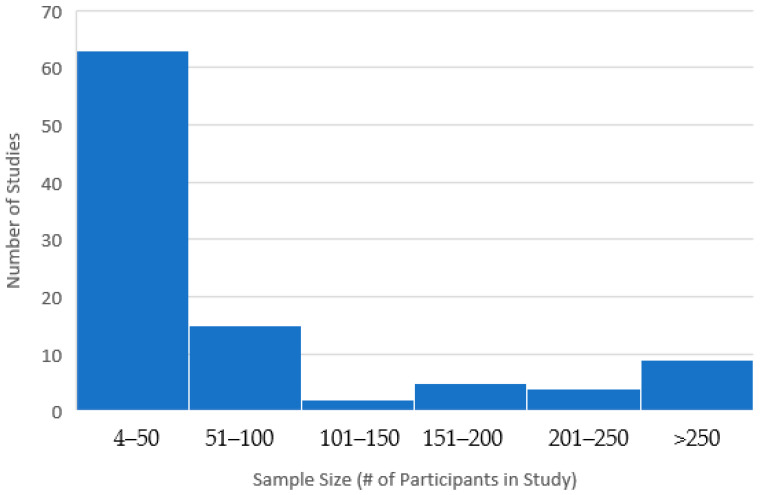
Histogram of sample sizes for included studies of long-acting therapeutics.

Sample sizes for the included studies ranged from 4 to 3457; 62% had a sample size under 50 individuals (78% had a sample size under 100) and only 9% had a sample size above 250 (See Figure 2). Because of a few large studies, however, the total number of individuals in the studies included in this review was 19,563.

Following full-text review, only three studies of long-term therapies were found to be completed solely in infants. Interestingly, in these studies focused on infants, there was no common theme. Investigators looked at treatments for various indications such as neonatal seizures, low B12 values, and West syndrome.

### 3.2. Type of Therapeutic Studied

The majority of studies of long-acting therapeutics in children and/or infants were for hormone therapies other than contraceptives, specifically treatments for precocious puberty. These studies were highly variable from one another looking at both individuals with male and female assigned genders at birth and various mechanisms of defining efficacy including, but not limited to, ovary size, LH and FSH levels, height velocity, and bone maturation. Five of the 46 studies found to be completed in children and infants also fell into the other category. These studies specifically looked at hyperinsulinism, nephrotic syndrome, Turner syndrome, and West syndrome.

Half of the studies of long-acting therapeutics in children and young people were found to have investigated the use of antibiotics (three out of six). However, all three of these studies looked at long-term intramuscular formulations of penicillin (Supplementary Table S1). In fact, all six studies looking at long-acting antibiotics that included children investigated the use of intramuscular penicillin. The indications under study for intramuscular penicillin were treatment of rhematic fever, yaws, or tonsilitis. Nonetheless, while these studies looked at a variety of indications, the use of long-acting intramuscular penicillin was found to be safe and efficacious in each study found.

The majority of studies of long-acting therapeutics in young people related to contraceptives. A significant portion of the studies in young people (14 of 40 studies) also investigated the use of other hormone therapies. Studies of long-term hormone therapies in this age group differed from those in children and infants because they looked at a variety of broader indications, including hirsutism, use of gonadotrophin-releasing hormone agonists in the treatment of lymphoma, congenital adrenal hyperplasia, and panhypopituitarism.

Clinical guidelines recommend consideration of long-acting therapeutics in non-pregnant adults for clinical indications as diverse as bipolar disorder I, schizophrenia, osteoporosis, hormonal treatment of hormone-responsive cancers, and many others. However, studies of long-acting therapeutics for these established indications in pregnant people and children are lacking.

One of the central findings of this review was that very minimal research on long-acting therapeutics has been performed among pregnant and postpartum people; we found that the entirety of the information published on long-acting therapeutics in pregnant people came from a total of 319 individuals. In pregnant individuals, there did not appear to be a common theme to the indications of long-acting therapeutics studied. Studies looked at the treatment of low sacral back pain, hyperemesis gravidarum, and the use of penicillin as an antibiotic. Surprisingly, in the studies which examined postpartum individuals, breastfeeding practices and breastmilk PKs were not investigated. All three of the studies in postpartum women were looking at contraceptive use. In the two studies in which contraceptive efficacy were investigated, both long-acting contraceptives were found to be efficacious.

### 3.3. Pharmacokinetic Content in Studies

Notably, despite the established potential for pharmacokinetic differences between children and adults, there was a particular dearth of studies which contained any pharmacokinetic characterization of drugs. This same dearth of pharmacokinetic information was also found in the studies with pregnant and postpartum individuals. Table 2 depicts the tabulation of pharmacokinetic content across the reviewed studies.

Of the reviewed studies, only 8.9% (9 out of 101—1 out of 6 of the studies in pregnancy and 8 out of 95 of the studies in children and adolescents) collected any pharmacokinetic data at all. More importantly, only four of the studies had an explicit and deliberate focus on pharmacokinetics and investigating potential changes in drug concentrations over time in a given special population. Moreover, the few studies that included PK data examined vastly different indications, making it difficult to draw any robust conclusions on the PKs of the long-acting therapeutic in special populations as compared to non-pregnant, non-postpartum adults.

### 3.4. Clinical Outcomes in Included Studies

The clinical outcomes with the long-acting therapeutics studied in the included articles were good overall. Full details of the results and conclusions of the included articles can be found in Supplemental Materials. For DMPA, biodegradable NET implant, and other long-acting hormonal formulations, safety concerns were minimal for the most part and contraceptive efficacy was high. Sustained reductions in schizophrenia symptoms and improvement in OCD and bipolar disorder were observed with long-acting antipsychotics, and sustained contraceptive efficacy was seen with long-acting reversible contraceptive forms like intrauterine devices. Long-acting parenteral formulations of testosterone and other hormones achieved growth and other targeted clinical outcomes in individuals with hypogonadotropic hypogonadism and other conditions. For long-acting benzathine penicillin, when used for long-term prophylaxis of rheumatic heart disease, there was some evidence of DNA damage as suggested by sister chromatid exchange analysis, but anaphylactic reactions were rare when compared to children without rheumatic fever not on long-acting benzathine penicillin. The use of benzathine penicillin in the third trimester, however, was well-tolerated and significantly reduced vaginal carriage of Group B streptococcus at delivery; its use for late latent syphilis in adolescence was similarly efficacious.

### 3.5. Route of Administration of Therapeutics

Finally, it is noteworthy that out of the 101 studies, the vast majority of long-acting therapeutics studied were delivered via the intramuscular route of administration. Two studies examined intrauterine devices, five studies examined subdermal implants, and the other 94 studies examined intramuscular injectable long-acting therapeutics.

## 4. Conclusions

There is inadequate information on long-acting therapeutics in special populations including infants, children, young people, and pregnant and postpartum people. This systematic review found a total of 101 articles pertaining to long-acting therapeutic strategies in these populations. There was a small pool of indications studied in these populations; hormonal contraception or hormone replacement therapy were the large majority (73/101 publications). This is unsurprising, as 89.7% of young people reported using some form of contraception according to the 2019 National Youth Risk Behavior Survey conducted by the CDC [25]. Other categories included antipsychotics and antibiotics. Notably, there were no studies of long-acting antiretrovirals or antivirals in special populations, despite a general call for such data on the part of the scientific and advocacy communities. This, however, is beginning to emerge between the historical period of our search (prior to 2018) and now; for example, PK and acceptability data on long-acting cabotegravir and rilpivirine in adolescents down to age 12 years have been presented and a study of these agents in children down to age 2 is now underway (IMPAACT 2036) [26].

The example of HIV provides a particularly potent illustration of why there is a need for more data on long-acting therapeutics in special populations. In many clinical settings, adolescence and the postpartum period are times when individuals are at particularly high risk for non-adherence to daily pills, and for falling out of care. At various times across the life cycle, there is a need for strategies to control HIV viral load that do not require as much user dependence or contact with the healthcare system. For example, a single administration of long-acting antiretrovirals at delivery, either for treatment or as PrEP, could provide protection both for the mother and her infant for a prolonged period.

Acceptability to patients of daily medication varies considerably from patient to patient and throughout different eras and contexts in a patient’s life. Unacceptability of medications is likely to impact adherence negatively. Existing studies on patient preferences and perspectives suggest that in many contexts, long-acting strategies are preferred over daily oral medications [27,28,29,30]. Parallel efforts to explore patient preferences around long-acting therapeutics in comparison to oral therapeutics should accompany specific research efforts for neglected populations.

Reversal of the research neglect of special populations with respect to long-acting therapeutics may be underway. On clinicaltrials.gov, there are 71 active studies listed investigating long-acting therapeutics for adolescents, many for the indication of asthma. HIV may lead the way; a specific trial of cabotegravir and rilpivirine in virally suppressed women with HIV (NCT 06336434) is now in the planning phases, 3 years after the drug’s initial approval. In addition, the HPTN 084 open label extension phase and PURPOSE 1 study of long-acting lenacapavir as PrEP (NCT 04994509) now allow women who become pregnant to remain in the study, and initial results showing favorable safety in pregnancy have been presented [31]. All of these studies will involve a component of pharmacokinetic assessment. However, there are many more areas within long-acting therapeutics that still demand research in special populations to ensure their broad and informed access for those who need them.

There were some limitations to our study. Firstly, foreign language articles were excluded, which might have provided some additional data. This review also covered only the years from 1980 through 2018. Therefore, clinical studies published from 2019 to the present were not included in this review. It is possible that the small average sample size of our included studies could have introduced bias, or that some design issues (such as the lack of a control group in many studies) could have introduced bias. A meta-analysis was not performed, largely because of extreme heterogeneity across study types and outcomes. The diversity of therapeutic areas represented by the studies in this review, and the heterogeneity of study designs, makes generalization challenging. However, it remains clear that there is a lack of deployment of long-acting strategies in research involving our focus populations. Although our search terms might have excluded some studies, we believe this review was quite comprehensive and unlikely to have missed any substantive publications related to the topic.

The main finding of our systematic review was that the long-acting therapeutics studied in children, youth, and pregnancy were largely well tolerated and efficacious. Long-acting hormonal therapeutics had some side effects (such as some loss of bone density among youth on DMPA, which was potentiated by lower calcium intake, higher alcohol intake, and lower BMI) but overall had beneficial clinical outcomes (e.g., prevention of chemotherapy-induced ovarian damage in young women with lymphoma with adjunctive long-acting gonadotrophin-releasing hormone agonist). When long-acting injectable antipsychotics were compared to oral versions of the same drug (e.g., risperidone), they were found to have superior control of schizophrenic symptoms. When long-acting injectable forms of HIV PrEP were compared to standard oral comparators, superiority in efficacy was often showna in the setting of pharmacokinetic substudies showing incomplete adherence to the oral comparator.

Looking to the future, any therapeutic area where patient clinical outcomes are stymied by non-adherence could potentially benefit from the use of longer acting therapeutics with less frequent dosing schedules. In addition, improving access of vulnerable populations to long-acting therapeutics has a high potential to improve important patient-centered outcomes such as quality of life and health inequities. In this review, we noted a favorable efficacy and tolerable safety profile of long-acting injectable antipsychotics in youth for indications from schizophrenia to bipolar disorder, and a similar safe and effective profile for long-acting contraception for the populations in whom it was studied. Long-acting formulations could similarly revolutionize the treatment and prevention of chronic infectious diseases such as HIV, tuberculosis, and hepatitis B and C virus infections. The benefits of improved HIV viral load suppression via increased adherence are not only advantageous for the individual patient but likely also extend to sexual partners and, in the case of pregnant people, their child, who may be at risk of acquiring the infection (potentially including children with perinatal exposure) [32]. More pharmacokinetic studies of long-acting agent, and more research on long-acting drug delivery technologies in specific areas such as bipolar disorder, depression, dyslipidemia, substance use disorders, and many other therapeutic areas are needed.

Long-acting therapeutics have high potential to benefit many. Infants, children, young people, and pregnant and postpartum women deserve safe and equitable access to these emerging technologies. Our systematic review revealed that across all therapeutic domains, from 1980 to 2018, there were a scant 101 articles relating to long-acting therapeutics in special populations. Moreover, most of these studies were lacking any pharmacokinetic characterization of the study drug, despite the potential for drug levels to vary across populations and to impact both efficacy and safety. Particularly because special populations can be especially vulnerable to experiencing adherence barriers and disruptions in care, long-acting therapeutics hold high potential to mitigate or circumvent these disruptions.

There are significant reasons to believe that long-acting formulations may have different PKs in childhood, adolescence, and pregnancy as compared to adulthood. For example, short-acting oral and parenteral drugs are well known to have different PK profiles in these populations [33,34]. As a consequence, their pharmacodynamics, toxicity, and efficacy could be different, and documenting this prior to widespread use of these formulations is important. Although this systematic review documents the significant knowledge gaps that exist, next steps need to include plans for additional work, some of which is already in progress.

## Figures and Tables

**Table 1:** Summary of study characteristics.

	Indication					
Special Population	Contraceptive	Antipsychotics	Antibiotic	Other Hormone Therapies	Other *	Total
Infants	N/A	N/A	N/A	0	3	3
Children ** and Infants	N/A	1	3	37	5	46
Children and Young People	0	0	3	1	2	6
Young People ***	18	3	4	14	1	40
Pregnant Women	N/A	0	1	0	2	3
Postpartum ****	3	0	0	0	0	3
Total	21	4	11	52	13	101

* Indications in the Other category included sickle cell disease, West syndrome, nephrotic syndrome, hyperinsulinism, Turner syndrome, asthma, vitamin B12 deficiency, sacral pain, hyperemesis gravidarum, acute lymphoblastic leukemia, and neonatal seizures. ** The age cutoff above which individuals were considered children was <15 years. *** Young people were defined by the WHO definition, which covers the age range of 10–24 years. **** Of these, two of the six studies reviewed were in postpartum young people. These were subtracted from the Young People category.

**Table 2:** Pharmacokinetic data in studies of long-acting therapeutics in special populations.

Population	PK Data Collected and Reported (n/N (%))	Hormone Levels Collected and Reported (n/N (%))
Infants, Children, and Young People (N = 95)	8 (9.3%)	6 (6.3%)
Pregnancy and Postpartum (N = 6)	1 (16.77%)	0 (0%)

## Supplementary Materials

The following supporting information can be downloaded at: https://www.mdpi.com/article/10.3390/pharmaceutics17010113/s1, Table S1: Table of Drug Formulations Studied Classified by Population and Indication; Table S2: PubMed Search Terms; Table S3: Cochrane Library Search Terms; Table S4: Embase Search Terms; Table S5: Details of Included Articles; PRISMA 2020 checklist.
