# Supplementary material for: Investigations of Long-Acting Formulations in Children, Adolescents, and Pregnant Women: A Systematic Review
Source: Pharmaceutics. 2025 Jan 15;17(1):113. doi: 10.3390/pharmaceutics17010113 (PMC11769521; doi:10.3390/pharmaceutics17010113)
Supplement: Supplementary file 1 [file pharmaceutics-17-00113-s001.zip › pharmaceutics-3268225-supplementary.pdf]

Supplementary Materials

Table S1. Table of Drug Formulations Studied Classified by Population and Indication

**Infants**	**Indication**
Phenobarbital *(lyophilized)*	Other: Neonatal Seizures
Synthetic adrenocorticotropic hormone (ACTH) (Tetracosactide (Synacthen))	Other: West Syndrome
Hydroxycobalamin (vitamin B12 depot)	Other: Low B12
**Children and/or infants**	**Indication**
Benzathine benzylpenicillin	Antibiotic
Benzathine Penicillin G	Antibiotic
Penicillin	Antibiotic
Risperidone	Antipsychotic
Decapeptyl	Other Hormone Therapy
Decapeptyl	Other Hormone Therapy
Decapeptyl	Other Hormone Therapy
Decapeptyl	Other Hormone Therapy
Decapeptyl	Other Hormone Therapy

Decapeptyl	Other Hormone Therapy
Depot GnRHa	Other Hormone Therapy
Depot leuprolide	Other Hormone Therapy
Depot leuprolide acetate	Other Hormone Therapy
Depot leuprolide acetate	Other Hormone Therapy
Depot-triptorelin	Other Hormone Therapy
GnRHa (long-acting D-Trp-6-GnRH)	Other Hormone Therapy
GnRHa triptorelin embonate	Other Hormone Therapy
Leuprolide acetate	Other Hormone Therapy
Leuprolide acetate	Other Hormone Therapy
Leuprolide acetate	Other Hormone Therapy
Leuprolide acetate depot	Other Hormone Therapy
LHRH	Other Hormone Therapy
LHRH	Other Hormone Therapy
Long acting GnRH analog (Decapeptyl Depot)	Other Hormone Therapy
Long-acting GnRH agonist triptorelin	Other Hormone Therapy
Long-acting GnRH agonist triptorelin	Other Hormone Therapy
Long-acting GnRH agonist triptorelin	Other Hormone Therapy
Long-acting GnRH agonist triptorelin depot (Decapeptyl-Depot)	Other Hormone Therapy
Long-acting GnRH analog (D-Trp6-GnRH)	Other Hormone Therapy

Long-Acting Gonadotropin-Releasing Hormone Analog<br><br>Leuprolide Acetate, Lupron Depot	Other Hormone Therapy
Testosterone depot; testosterone propionate and testosterone enenthate	Other Hormone Therapy
Triptorelin	Other Hormone Therapy
Triptorelin	Other Hormone Therapy
Triptorelin	Other Hormone Therapy
Triptorelin	Other Hormone Therapy
Triptorelin	Other Hormone Therapy
Triptorelin	Other Hormone Therapy
Triptorelin	Other Hormone Therapy
Triptorelin	Other Hormone Therapy
Triptorelin	Other Hormone Therapy
Triptorelin depot	Other Hormone Therapy
Long-acting release (LAR) octreotide (Sandostatin LP, Novartis)	Other: Hyperinsulinism
Triamcinolone acetonide	Other: Nephrotic Syndrome
Depot estradiol	Other: Turner's Syndrome
Long acting synthetic ACTH	Other: West Syndrome
Synthetic depot ACTH	Other: West Syndrome
**Children and Young People**	**Indication**

Benzathine Penicillin	Antibiotic
Benzathine Penicillin G	Antibiotic
Long-acting penicillin	Antibiotic
GnRH agonist D-Trp6-LHRH, depot preparation	Other Hormone Therapy
PEG-asparaginase	Other: Acute lymphoblastic leukemia
Dexamethasone	Other: Asthma
**Young People**	**Indication**
Benzathine penicillin	Antibiotic
Benzathine penicillin G	Antibiotic
Benzathine Penicillin G	Antibiotic
Tifomycine	Antibiotic
LAIR	Antipsychotic
Long-acting risperidone	Antipsychotic
Long-acting injectable risperidone	Antipsychotic
Biodegradable norethisterone (NET) implant	Contraceptive
Cyclofem	Contraceptive
Depo-Provera	Contraceptive
Depo-Provera	Contraceptive
Depot medroxyprogesterone acetate (DMPA)	Contraceptive
Depot medroxyprogesterone acetate (DMPA)	Contraceptive

Depot-medroxyprogesterone acetate (DMPA)	Contraceptive
DMPA	Contraceptive
DMPA	Contraceptive
DMPA NET-EN	Contraceptive
Levonorgestrel intrauterine device (LNG IUD)	Contraceptive
Norethisterone oenanthate	Contraceptive
Norethisterone oenanthate	Contraceptive
Perlutal+Dihydroxyprogesterone Acetophenide	Contraceptive
Progesterone vaginal ring	Contraceptive
Progestin levonorgestre	Contraceptive
Uniplant	Contraceptive
Uniplant	Contraceptive
Decapeptyl	Other Hormone Therapy
Decapeptyl	Other Hormone Therapy
Depot D-TRP6-GnRH-a	Other Hormone Therapy
Depot D-TRP6-GnRH-a	Other Hormone Therapy
Depot testosterone	Other Hormone Therapy
Depot testosterone esters	Other Hormone Therapy
Long acting GnRH analog (Decapeptyl Depot)	Other Hormone Therapy
Long-acting D-Trp6-luteinizing hormone-releasing hormone	Other Hormone Therapy
Long-acting T preparation	Other Hormone Therapy

Nebid	Other Hormone Therapy
Progynonm Depot	Other Hormone Therapy
SUS 100	Other Hormone Therapy
T enanthate	Other Hormone Therapy
Triptoreline	Other Hormone Therapy
Piroxicam	Other: Sickle Cell
**Pregnant**	**Indication**
Benzathine penicillin G	Antibiotic
Diprofos Schering Plough	Other: Hyperemesis Gravidarum
Triamcinolone	Other: Sacral low back pain
**Postpartum**	**Indication**
Etonogestrel-releasing contraceptive implant	Contraceptive
Subdermal Silastic implant	Contraceptive
Norethisterone enanthate	Contraceptive

Table S2. PubMed Search Terms

#1	"Pregnancy"[Mesh] OR "Breast Feeding"[Mesh] OR "Maternal Exposure"[Mesh] OR "Pregnancy Complications"[Mesh] OR "Abnormalities, Drug-Induced"[Mesh]	
#2	maternal[tw] OR maternity[tw] OR mother*[tw] OR pregnant*[tw] OR pregnanc*[tw] OR breastfeeding*[tw] OR breast feed*[tw] OR MTCT[tw] OR "vertical transmission"[tw]	
#3	"Infant"[Mesh] OR "Child"[Mesh] OR "Adolescent"[Mesh] OR "Minors"[Mesh] OR "Puberty"[Mesh] OR "Adolescent Health"[Mesh] OR "Child Health"[Mesh] OR "Infant Health"[Mesh] OR "Pediatrics"[Mesh] OR "Young Adult"[Mesh]	
#4	infant*[tw] OR infanc*[tw] OR baby[tw] OR babies[tw] OR toddler*[tw] OR child*[tw] OR adolescen*[tw] OR teen*[tw] OR youth*[tw] OR young adult*[tw] OR young people*[tw] OR juvenile*[tw] OR pediatric*[tw] OR paediatric*[tw] OR preschool*[tw] OR pre school*[tw] OR "under 5"[tw] OR under five*[tw] OR "less than five"[tw] OR newborn*[tw] OR new born*[tw] OR neonate*[tw] OR prenatal[tw]	
#5	#1 OR #2 OR #3 OR #4	

#6	"Delayed-Action Preparations"[Mesh:NoExp] OR "Injections, Intramuscular"[Mesh] OR "Intramuscular Absorption"[Mesh]	
#7	"long acting"[tw] OR longacting[tw] OR prolonged release[tw] OR prolonged action[tw] OR controlled release[tw] OR timed release[tw] OR sustained release[tw] OR delayed action[tw] OR depot preparation*[tw] OR depot medication*[tw] OR depot inject*[tw] OR Intramuscular*[tw] OR Intra muscular*[tw] OR "IM administration"[tw]	
#8	"GSK1265744"[nm] OR Cabotegravir[tw] OR GSK1265744*[tw] OR GSK 1265744*[tw] OR GSK744*[tw] OR GSK 744*[tw] OR "1051375-10-0"[rn]	
#9	"Rilpivirine"[Mesh] OR Rilpivirine[tw] OR "R278474"[tw] OR "R 278474"[tw] OR "TMC278"[tw] OR "TMC 278"[tw] OR edurant[tw] OR "500287-72-9"[rn] OR "700361-47-3"[rn]	
#10	"Paliperidone Palmitate"[Mesh] OR paliperidone[tw] OR "9 OH risperidone"[tw] OR "9 Hydroxy risperidone"[tw] OR "9 Hydroxyrisperidone"[tw] OR Invega[tw] OR "R 76477"[tw] OR R76477[tw] OR "ro 76477"[tw] OR "ro 92670"[tw] OR ro76477[tw] OR ro92670[tw] OR trevicta[tw] OR xeplion[tw] OR "144598-75-4"[rn] OR "199739-10-1"[rn]	
#11	#6 OR #7 OR #8 OR #9 OR #10	
#12	#5 AND #11	
#13	("Clinical Trial"[pt] OR "Clinical Trials as Topic"[mesh] OR "Epidemiologic Research Design"[Mesh] OR "Research Design"[mesh:noexp] OR "Early	

	Termination of Clinical Trials"[Mesh] OR "Comparative Study"[pt] OR "Evaluation Studies"[pt] OR "Multicenter Study" [pt] OR "Validation Studies" [pt] OR "Follow-up Studies"[mesh] OR "Prospective Studies"[mesh] OR Drug Therapy[sh] OR "Epidemiologic Studies"[Mesh] OR clinical trial*[tw] OR ((singl*[tw] OR doubl*[tw] OR trebl*[tw]) AND (mask*[tw] OR blind*[tw])) OR placebo*[tw] OR random*[tw] OR control[tw] OR controls[tw] OR prospectiv*[tw] OR volunteer*[tw] OR "Phase One"[tw] OR "Phase 1"[tw] OR "Phase I"[tw] OR "Phase Two"[tw] OR "Phase 2"[tw] OR "Phase II"[tw] OR "Phase Three"[tw] OR "Phase 3"[tw] OR "Phase III"[tw] OR "Phase Four"[tw] OR "Phase 4"[tw] OR "Phase IV"[tw] OR "4 arm"[tw] OR "four arm"[tw] OR RCT[tw] OR CCT[tw] OR multicenter[tw] OR multicentre[tw] OR trial[tw] OR trials*[tw] OR groups[tw] OR "latin square"[tw] OR clinical stud*[tw]) NOT (animals[mh] NOT humans[mh])	
#14	#12 AND #13	
#15	Filters: Publication date from 1980/01/01	

Table S3. Cochrane Library Search Terms

#1 MeSH descriptor: [Pregnancy] explode all trees

#2 MeSH descriptor: [Breast Feeding] explode all trees

#3 MeSH descriptor: [Maternal Exposure] explode all trees

#4 MeSH descriptor: [Pregnancy Complications] explode all trees

#5 MeSH descriptor: [Abnormalities, Drug-Induced] explode all trees

#6 maternal or maternity or mother* or pregnant* or pregnanc* or breastfeeding* or (breast next/1 feed*) or MTCT or "vertical transmission"

#7 MeSH descriptor: [Infant] explode all trees

#8 MeSH descriptor: [Child] explode all trees

#9 MeSH descriptor: [Adolescent] explode all trees

#10 MeSH descriptor: [Minors] explode all trees

#11 MeSH descriptor: [Puberty] explode all trees

#12 MeSH descriptor: [Adolescent Health] explode all trees

#13 MeSH descriptor: [Child Health] explode all trees

#14 MeSH descriptor: [Infant Health] explode all trees

#15 MeSH descriptor: [Pediatrics] explode all trees

#16 MeSH descriptor: [Young Adult] explode all trees

#17 infant* or infanc* or baby or babies or toddler* or child* or adolescen* or teen* or youth* or (young next/1 adult*) or (young next/1 people*) or juvenile* or pediatric* or

paediatric* or preschool* or (pre next/1 school*) or "under 5" or (under next/1 five*) or "less than five" or newborn* or (new next/1 born*) or neonate* or prenatal

#18 6-#17

#19 MeSH descriptor: [Delayed-Action Preparations] this term only

#20 MeSH descriptor: [Injections, Intramuscular] explode all trees

#21 MeSH descriptor: [Intramuscular Absorption] explode all trees

#22 "long acting" or longacting or "prolonged release" or "prolonged action" or "controlled release" or "timed release" or "sustained release" or "delayed action" or (depot next/1 preparation*) or (depot next/1 medication*) or (depot next/1 inject*) or Intramuscular* or (Intra next/1 muscular*) or "IM administration"

#23 Cabotegravir or GSK1265744* or "GSK 1265744" or "GSK744" or "GSK 744" or "1051375-10-0"

#24 MeSH descriptor: [Rilpivirine] explode all trees

#25 Rilpivirine or "R278474" or "R 278474" or "TMC278" or "TMC 278" or edurant or "500287-72-9" or "700361-47-3"

#26 MeSH descriptor: [Paliperidone Palmitate] explode all trees

#27 paliperidone or "9 OH risperidone" or "9 Hydroxy risperidone" or "9 Hydroxyrisperidone" or Invega or "R 76477" or R76477 or "ro 76477" or "ro 92670" or ro76477 or ro92670 or trevicta or xeplion or "144598-75-4" or "199739-10-1"

#28 6-#27

#29 #18 and #28

#30 #29 Publication Year from 1980

Table S4. Embase Search Terms

#1	'pregnancy'/exp OR 'breast feeding'/exp OR 'maternal exposure'/exp OR 'pregnancy complication'/exp OR 'drug induced malformation'/exp
#2	(maternal OR maternity OR mother* OR pregnant* OR pregnanc* OR breastfeeding* OR "breast feed*" OR MTCT OR "vertical transmission"):ab,ti,kw
#3	'infant'/exp OR 'juvenile'/exp OR 'minor (person)'/exp OR 'puberty'/exp OR 'adolescence'/exp OR 'childhood'/exp OR 'newborn period'/exp OR 'adolescent health'/exp OR 'child health'/exp OR 'pediatrics'/exp OR 'young adult'/exp
#4	(infant* OR infanc* OR baby OR babies OR toddler* OR child* OR adolescen* OR teen* OR youth* OR "young adult*" OR "young people*" OR juvenile* OR pediatric* OR paediatric* OR preschool* OR "pre school*" OR "under 5" OR "under five*" OR "less than five" OR newborn* OR "new born*" OR neonate* OR prenatal):ab,ti,kw
#5	#1 OR #2 OR #3 OR #4
#6	'delayed release formulation'/exp OR 'intramuscular drug administration'/exp OR 'intramuscular absorption'/exp
#7	("long acting" OR longacting OR "prolonged release" OR "prolonged action" OR "controlled release" OR "timed release" OR "sustained release" OR "delayed action" OR "depot preparation*" OR "depot medication*" OR "depot inject*" OR Intramuscular* OR "Intra muscular*" OR "IM administration"):ab,ti,kw

#8	'cabotegravir'/exp OR (Cabotegravir OR GSK1265744* OR "GSK 1265744*" OR GSK744* OR "GSK 744*" OR "1051375-10-0"):ab,ti,kw,tn
#9	'rilpivirine'/exp OR (Rilpivirine OR "R278474" OR "R 278474" OR "TMC278" OR "TMC 278" OR edurant OR "500287-72-9" OR "700361-47-3"):ab,ti,kw,tn
#10	'paliperidone'/exp OR (paliperidone OR "9 OH risperidone" OR "9 Hydroxy risperidone" OR "9 Hydroxyrisperidone" OR Invega OR "R 76477" OR R76477 OR "ro 76477" OR "ro 92670" OR ro76477 OR ro92670 OR trevicta OR xeplion OR "144598-75-4" OR "199739-10-1"):ab,ti,kw,tn
#11	#6 OR #7 OR #8 OR #9 OR #10
#12	#5 AND #11
#13	'clinical trial'/exp OR 'clinical trial (topic)'/exp OR 'epidemiology'/exp OR 'methodology'/exp OR 'early termination of clinical trial'/exp OR 'comparative study'/exp OR 'evaluation study'/exp OR 'multicenter study (topic)'/exp OR 'validation study'/exp OR 'follow up'/exp OR 'prospective study'/exp OR 'drug therapy'/exp
#14	("clinical trial*" OR ((singl* OR doubl* OR trebl*) AND (mask* OR blind*)) OR placebo* OR random* OR control OR controls OR prospectiv* OR volunteer* OR "Phase One" OR "Phase 1" OR "Phase I" OR "Phase Two" OR "Phase 2" OR "Phase II" OR "Phase Three" OR "Phase 3" OR "Phase III" OR "Phase Four" OR "Phase 4" OR "Phase IV" OR "4 arm" OR "four arm" OR RCT OR CCT OR multicenter OR multicentre OR trial OR trials* OR groups OR "latin square" OR clinical stud*):ab,ti,kw
#15	#13 OR #14

#16	#12 AND #15
#17	('animal'/exp NOT 'human'/exp)
#18	#16 NOT #17
#19	#18 AND [1980-2018]/py
